# Investigations on the preparation of ceramsite from petrochemical excess sludge

**DOI:** 10.3389/fchem.2022.1008884

**Published:** 2022-09-15

**Authors:** Zhengwei Liu, Hai Zhang, Xiaoyu Lin, Hongzhe Zhang, Zhiyuan Zhang, Shucai Zhang

**Affiliations:** ^1^ State Key Laboratory of Safety and Control for Chemicals, SINOPEC Research Institute of Safety Engineering Co. Ltd, Qingdao, China; ^2^ National Registration Center for Chemicals of the Ministry of Emergency Management of the People’s Republic of China, Qingdao, China

**Keywords:** excess sludge, ceramsite, fluxing agent, sintering behavior, leaching characteristics

## Abstract

Petrochemical excess sludge, as hazardous solid waste, poses a great threat to the environment and is difficult to dispose of in an economic and environmentally friendly way. A new alternative of using the petrochemical excess sludge to prepare ceramsite is proposed. The relationship between the sintering behavior of dried excess sludge, including the composition, temperature, fluxing agent, and pore-forming agent addition, and the properties of ceramsite is investigated. The properties of ceramsite are primarily affected by the sintering temperature and the addition of a fluxing agent. Ceramsite with a sintering-expanded surface is prepared. Also, its water absorption is quite low, indicating an improvement in densification due to sintering. Moreover, the leaching toxicity of the heavy metals in the dried excess sludge and prepared ceramsite is also investigated. It reveals the feasibility of ceramsite preparation by sintering petrochemical excess sludge.

## Introduction

The petrochemical industry brings about a great deal of energy and convenience for the development of human beings. Meanwhile, the petrochemical industry also produces a large quantity of solid waste, which induces a great environmental hazard to human beings if not properly disposed of (Abouelnasr and Isam, 2008; Elektorowicz and Habibi, 2005; Heidarzadeh et al., 2010; Begum et al., 2013; Hu et al., 2015; Qin et al., 2017; Fabio et al., 2018; Moško et al., 2018). For instance, an annual production of more than one million tons of petrochemical oily waste from a variety of sources, including crude oil tank bottoms, oil/water separators, and on-site wastewater treatment plants, is generated by the petrochemical industry in China (Hu et al., 2013). Among the solid oily waste produced, the excess sludge as an inevitable by-product from the petrochemical on-site wastewater treatment plant accounts for the largest portion. For instance, a ten-million-ton oil refinery plant produces about 10,000 m^3^/a excess sludge, and a one-million-ton ethylene plant produces more than 40,000 m^3^/a excess sludge.

The petrochemical excess sludge is a complicated mixture containing different quantities of waste mineral oil, such as benzene, xylene, pyrene, and petroleum hydrocarbon; heavy metals such as Pb, V, and Ni; and mineral matter. Some of the chemicals are toxic, carcinogenic, and mutagenic. Therefore, excess sludge is referred to as one kind of hazardous solid waste in most cases (Guo et al., 2019). Due to its adverse nature, a variety of methods have been applied to treat petrochemical excess sludge, such as thermal drying, pyrolysis, incineration, or landfills at hazardous waste sites (Elektorowicz and Habibi, 2005; Hu et al., 2013; Guo et al., 2019). However, these technologies have problems such as secondary pollution, especially the disposal of residue fly ash after incineration, and land occupation of the landfill treatment.

A similar occurrence is that excess sludge in the municipal wastewater treatment plant is becoming a critical environmental threat due to the treatment required for the enormous increase in the volume of wastewater (Bruno et al., 2022). Among the various treatment approaches for municipal excess sludge, ceramsite prepared from excess sludge, a kind of lightweight aggregate, has been reported and commercialized (Furlani et al., 2011; Li et al., 2018; Liao and Huang, 2012; Wang et al., 2008). For instance, Wang et al. investigated the sintering behavior of dried excess sludge and the related sintering mechanisms (Wang et al., 2008).

In contrast, the ignition loss ratio of the petrochemical excess sludge is between 80% and 90%. This is much higher than that of municipal excess sludge, which is between 40% and 70%. In other words, the content of ash after thermal incineration of the petrochemical excess sludge, which is expected to form the main crystalline matter of the ceramsites, is lower than that of municipal excess sludge. In addition, the content of alkali and alkaline-earth metals in the petrochemical excess sludge is lower than that of municipal excess sludge. The main function of the alkali and alkaline-earth metals during the preparation of ceramsites is to reduce the melting point and thus to prepare the targeted product at a relatively low temperature (Wang et al., 2008).

However, there have been no reports about the preparation of ceramsite from the petrochemical excess sludge. If the petrochemical excess sludge can be used to prepare the ceramsite, the excess sludge can be comprehensively utilized and the ash that would be produced from the incineration of excess sludge is solidified to be the component of ceramsites, which is finally vitrified at ceramsite. The amount of ash or heavy metal that can be extracted from the environment can be dramatically decreased. In addition, the high content of organic matters that would vaporize at a high temperature can generate many pores and be beneficial to the preparation of ceramsites during sintering.

Therefore, the aim of this work is to study the sintering characteristics and behavior of excess sludge produced from the petrochemical wastewater plant under varying sintering conditions, such as different compositions and temperatures. Also, during the sintering, reactions and transformations occur between the silicate and the associated minerals, which result in the formation of various crystalline phases. All, of these transformations and products play a critical role in the properties of ceramsite (Moško et al., 2018; Wang et al., 2008). Thus, the phase and microstructure transformations are also investigated using X-ray diffraction and scanning electron microscopy. Moreover, the leaching behavior of typical heavy metals is included in this article to discuss the environmental safety of the as-prepared products.

## Materials and methods

### Synthesis procedure

The petrochemical excess sludge used in this study was acquired from a petrochemical wastewater treatment plant in Qingdao, China. The excess sludge was first dewatered mechanically via plate-frame pressure filtration, dried in a baking oven at a temperature of 85°C until the weight did not change, and then powdered with a ball mill until it could pass through a 150 mesh sieve. The clay was bought from Shandong Haoyao Co., Ltd. The fluxing agent Na_2_O (in the form of Na_2_CO_3_, analytical grade) and pore-forming agent Fe_2_O_3_ were bought from Sinopharm Chemical Reagent Co., Ltd. The powdered excess sludge, clay, and other additions were first thoroughly blended. Spherical specimens with a diameter of around 20 mm were prepared by wet rolling, dried, and subsequently sintered in an electric kiln with sintering temperatures ranging from 1,150 to 1200°C for 15 min. A ramp rate of 15 ^°^C/min and temperature preservation at 350°C for 15 min were used.

### Characterization

The major chemical compositions of excess sludge and the clay, including Si, Al, Ca, Fe, Mg, K, Na, and P, were analyzed using an X-ray fluorescence spectrometer (XRF, Thermo Fisher Scientific ARL QUANT'X EDXRF) after sintering at 1200°C for 15 min, and expressed in the form of oxide. X-ray diffraction (XRD, Bruker D8 Advance) using 40 mA and 40 kV Cu Kα radiation was used for the research of crystalline phases in sintered products. The crystalline phases were examined by comparing the positions of the Bragg peaks with the data files of the Joint Committee on Powder Diffraction Standards (JCPDS). The microstructural morphology was recognized using a scanning electron microscope (SEM, JSM-6460LV).

The leaching toxicity of trace hazardous elements, including Ni, Cd, Pb, Cr, Cu, and V, in excess sludge and sintered product was determined by horizontal vibration extraction procedure (HVEP) according to HJ/T 299-2007 and GB 5085.3-2007 of China (GB5085, 2007; HJ/T299, 2007; Chen et al., 2022). The chosen samples were roughly crushed and pulverized in a laboratory grinding machine for 5 min and then dried in an oven at 80°C until reaching a constant weight. Leaching of the heavy metals: 150 g of pulverized ceramsite was placed in a Teflon bottle. Then, an acid solution with a pH of 3.2 (configured by a mixture of concentrated sulfuric acid and concentrated nitric acid with a mass ratio of 2:1) was added into the Teflon bottle to maintain the liquid–solid ratio (L:kg) of 10:1. The bottle was then fixed in a flip-type shaker and shaken at a speed of 30 r/min for 20 h. After the oscillation, the leachate was collected and stored under cold conditions. The elemental concentration was measured by inductively coupled plasma mass spectrometry (ICP-MS, Thermo Fisher iCAP 7000).

The values of 24 h water absorption and bulk density were obtained as follows:

$$24\;h\;\text{water}\;\text{abosorption}\;\text{rate}=\frac{24\;h\;\text{saturated}\;\text{weight}-\text{dry}\;\text{weight}\;\text{of}\;\text{ceramsite}}{\text{dry}\;\text{weight}\;\text{of}\;\text{ceramsite}}$$ (2.1)

$$\text{bulk}\;\text{density}=\frac{\text{weight}\;\text{of}\;\text{ceramsite}}{\text{volume}\;\text{of}\;\text{ceramsite}}$$ (2.2)

## Results and Discussion

### Properties of the raw material

According to the study conducted by Riley (Riley, 2006), the theoretical composition to prepare the ceramsite is SiO_2_, 55%–77%;、A1_2_O_3_, 10%–25%;、other compositions (including K_2_O、CaO、Fe_2_O_3_、MgO、Na_2_O), 8%–25%. XRF was conducted to analyze the chemical composition of the excess sludge after sintering at 1200°C for 15 min as shown in Table 1. The content of Al_2_O_3_ is 41.6%, relatively higher than that of SiO_2_, which is 28.6% in excess sludge. This is contrary to the theoretical demand of SiO_2_ being much higher than that of Al_2_O_3_ if the material is expected to be used to prepare the sintering-expanded ceramsite (Riley, 2006; Liao and Huang, 2012). The total content of ceramsite structure-forming agents in the excess sludge, mainly including SiO_2_ and Al_2_O_3_, reaches 70.2%, which is also not sufficient to prepare the sintering-expanded ceramsite (Liao and Huang, 2012). More importantly, the total content of the alkali and alkaline-earth oxide in excess sludge, including CaO, MgO, K_2_O, and Na_2_O, is lower than that in the clay and municipal sludge (Wang et al., 2008). Especially, the key fluxing agent of K_2_O and Na_2_O is not detected, which could influence the melting point considerably. It suggests a higher melting point if the excess sludge is meant to be used to prepare the sintering-expanded ceramsite.

**TABLE 1:** Chemical composition of excess sludge produced in a petrochemical wastewater treatment plant and clay after sintering at 1200°C for 15 min (wt%).

	SiO_2_	Al_2_O_3_	CaO	MgO	K_2_O	Na_2_O	Fe_2_O_3_	P_2_O_5_
Excess sludge	28.6	41.6	0	6.5	0	0	8.3	15
Clay	63.12	27	1.67	1.16	1.3	3.5	2.25	0

**FIGURE 1:**
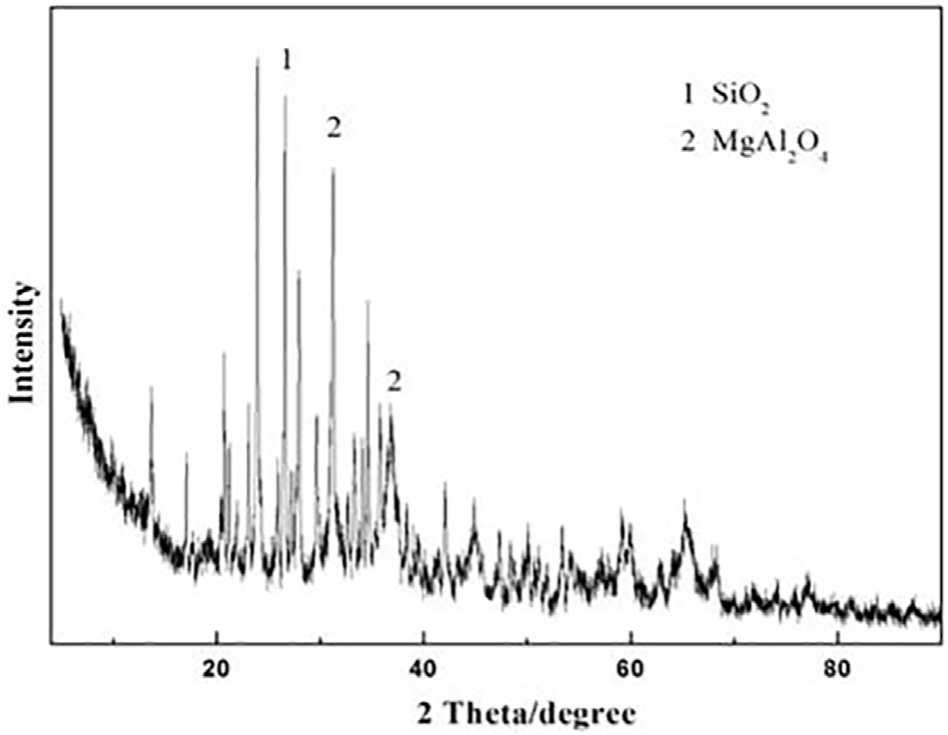
XRD pattern of the excess sludge after sintering at 1200°C for 15 min.

**FIGURE 2:**
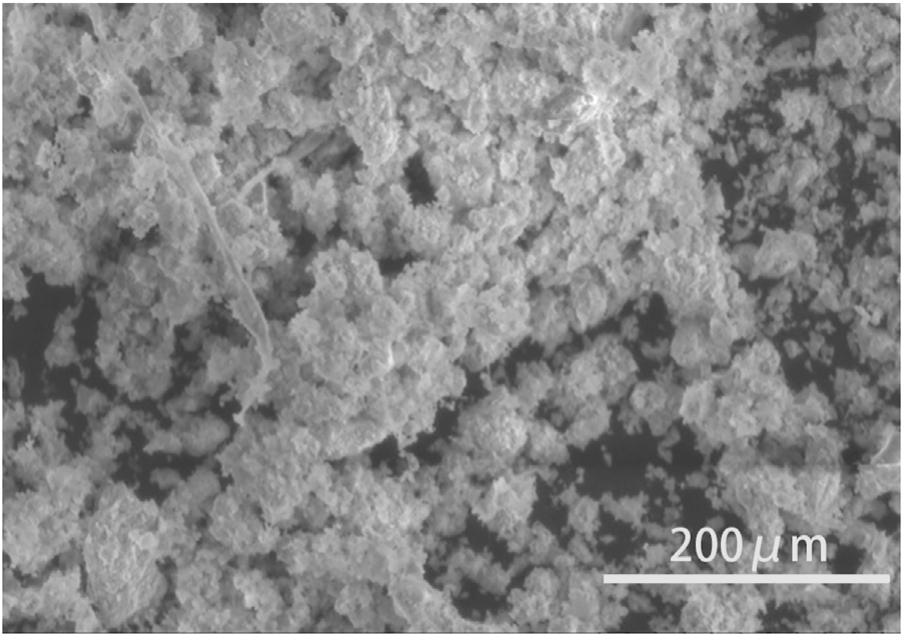
SEM image of the excess sludge after sintering at 1200°C for 15 min.

The XRD pattern of the sintered excess sludge at 1200°C for 15 min is displayed in Figure 1. The main diffraction peak is attributed to SiO_2_ and MgAl_2_O_4_ phases, while some peaks cannot be identified accurately. The SEM morphology of the sintered excess sludge at 1200°C for 15 min is displayed in Figure 2, which consists of many 30 μm spherical particles.

The ignition loss of the dried excess sludge after sintering at 1200°C for 15 min is as high as 86%. That is to say, the easily volatile component of the dried excess sludge treated at elevated temperatures is quite high. This implies that the excess sludge cannot be individually employed to prepare the sintering-expanded ceramsite due to the large ignition loss ratio of the petrochemical excess sludge.

### Influence of clay addition on the property of ceramsite

Due to the high content of the easily volatile component in excess sludge as aforementioned, the excess sludge cannot be individually utilized to prepare ceramsite. Some materials comprising high content of SiO_2_ and Al_2_O_3_ must be supplemented in order to regulate the chemical composition of the excess sludge. In this study, clay was selected because of its high content of ceramsite structure-forming composition and its wide usage in ceramsite preparation. Therefore, the mixture containing 80 wt% clay and 20 wt% dried excess sludge was first employed to prepare the ceramsite at a sintering temperature of 1200°C.

**FIGURE 3:**
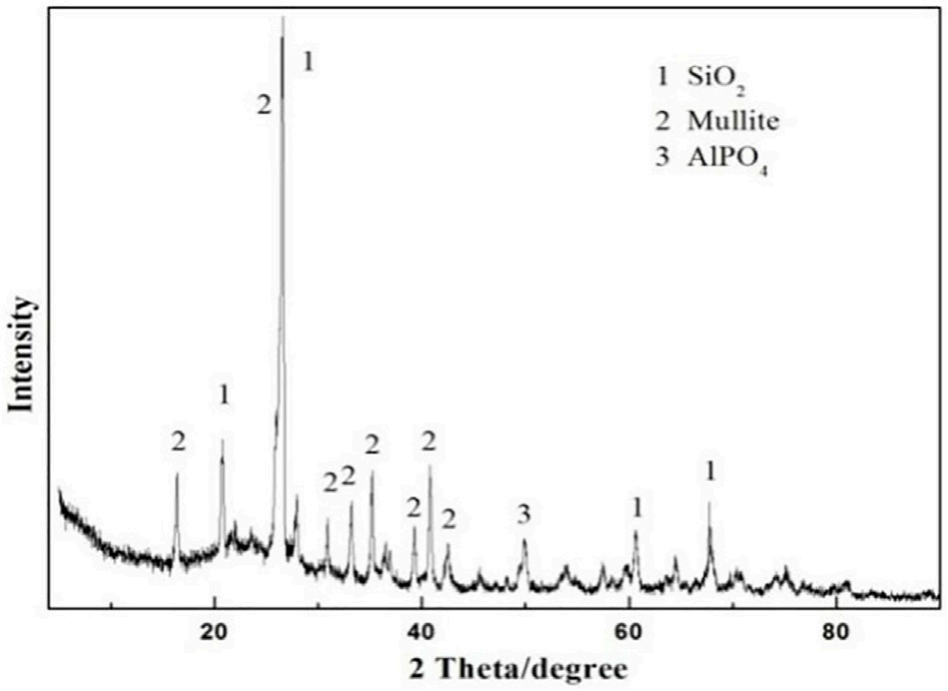
XRD pattern of the prepared ceramsite made of 80 wt% clay and 20 wt% dried excess sludge at a sintering temperature of 1200°C.

The as-prepared product has a solid outer surface. However, the ceramsite does not show any evidence of surface melting or volume expansion. That is to say, the sintering-expanded ceramsite is not prepared. This is mainly because the sintering temperature is too low to make the ceramsite crystal phase molten due to the low content of the alkali and alkaline-earth oxide in excess sludge, which primarily functions in reducing the melting point (Wang et al., 2008). The XRD pattern in Figure 3 displays the main crystalline phase including quartz (SiO_2_), mullite, and aluminum phosphate (AlPO_4_). This is quite different from the original phase of the excess sludge as shown in Figure 1, indicating some kinds of chemical changes have occurred.

### Influence of the fluxing agent on the property of ceramsite

Due to the nonmelting surface as verified above, another chemical agent must be supplemented to the mixture of excess sludge and clay in order to reduce the melting temperature and thus prepare the sintering-expanded ceramsite. Therefore, the influence of the fluxing agent Na_2_O (in the form of Na_2_CO_3_) on the property of ceramsite was investigated by the addition of different contents of fluxing agent Na_2_O to the mixture of clay and excess sludge. The content of the dried excess sludge is 20 wt%. The fluxing agent is 5 wt%, 10 wt%, and 15 wt%, respectively. The remaining is clay. Then, the mixture is subject to sintering at 1200°C for 15 min.

**FIGURE 4:**
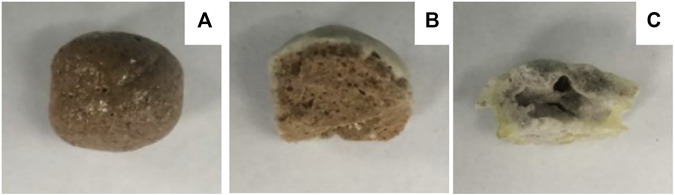
Image of the prepared ceramsite with different contents of **(A)** 5%, **(B)** 10%, and **(C)** 15% Na_2_O at 1200°C for 15 min.

The image of the prepared ceramsites with different contents of fluxing agent Na_2_O is displayed in Figure 4, showing a completely different surface and cross-sectional image. The ceramsite prepared with a 5% fluxing agent shows a melting surface and hard cover. The ceramsite prepared with a 10% fluxing agent shows a white cover, while the cross-section of the ceramsite shows the formation of bubbles, same as the ceramsite prepared with a 5% fluxing agent. It is noted that the ceramsite prepared with a 15% fluxing agent, which is supposed to decrease the melting temperature most significantly, shows a completely different color. Also, it does not show evidence of the melting phenomenon and bubble formation. In summary, the mixture with the addition of 10% and 15% fluxing agents fails to prepare the ceramsite.

**FIGURE 5:**
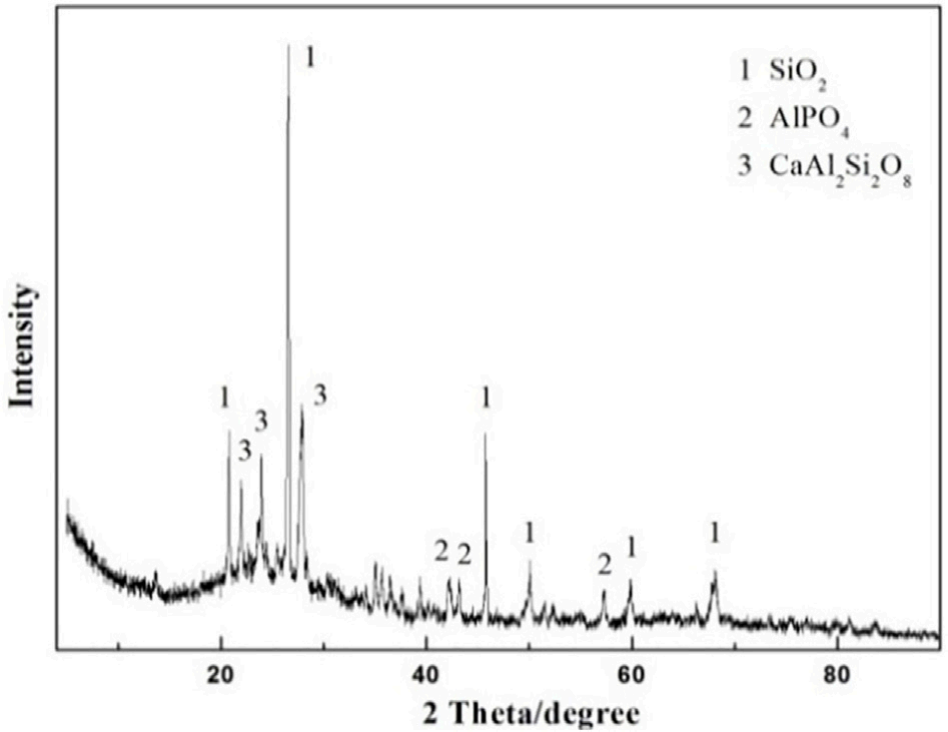
XRD pattern of the prepared ceramsite with the addition of 5 wt% Na_2_O at 1200°C for 15 min.

The XRD pattern of the as-prepared ceramsite with the addition of a 5% fluxing agent is displayed in Figure 5. The principal crystalline diffraction peaks identified in the prepared ceramsite are attributed to SiO_2_, AlPO_4_, and anorthite (CaAl_2_Si_2_O_8_, i.e., CaO·Al_2_O_3_·2SiO_2_), which indicated that some reaction occurred. As displayed above, the diffraction peak of the prepared ceramsite with the addition of fluxing agent Na_2_O is also dissimilar to that of the prepared ceramsite without the addition of fluxing agent Na_2_O identified in Figure 3. The mullite phase is recognized in the prepared ceramsite without the addition of fluxing agent Na_2_O, while the CaAl_2_Si_2_O_8_ phase is identified in the prepared ceramsite with the addition of fluxing agent Na_2_O, whose melting point is about 1130°C. This is the indication of chemical reactions occurring during the sintering of the mixed raw material. It is speculated that the addition of a slight amount of fluxing agent changes the chemical reactions and can noticeably reduce the melting temperature compared with that without the addition of fluxing agent, which can thus effectively improve the ceramsite quality and lower the sintering temperature.

**FIGURE 6:**
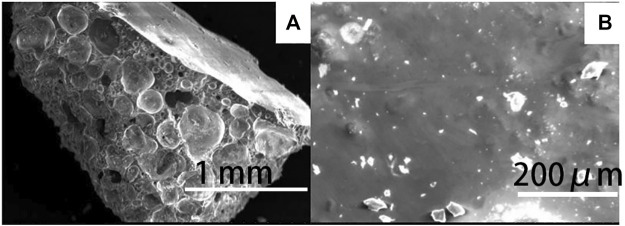
SEM image of **(A)** the cross-section and **(B)** the outer surface of the prepared ceramsite with the addition of 5 wt% Na_2_O at 1200°C for 15 min.

Figure 6A shows the microstructure of the cross-section of the prepared ceramsite with the addition of 5 wt% Na_2_O examined by SEM. Bubbles are generated with the addition of fluxing agent Na_2_O, and the diameter of the large bubble is about 0.4 mm. The bubble is relatively separate from each other and is not interconnected. The surface of the bubble is relatively smooth. Therefore, the existence of the bubble indicates that melting occurs and the bubble expands to increase the size of the ceramsite due to the bloating effect (Riley, 2006). Such bloating effect causes the lightweight property of the ceramsite, and the bulk density of the ceramsite is about 1.06 g/cm^3^. The outer surface of the ceramsite is shown in Figure 6B, which displays a dense melting phenomenon to cover the whole surface. This is why the 24 h water absorption rate is quite low at about 1.4%. The prepared ceramsite could be used as construction material. Compared with the relatively low ceramsite preparation temperature of 1080°C from the municipal excess sludge, the high preparation temperature of 1200°C from petrochemical excess sludge can be attributed to the low content of the fluxing agent, including CaO, MgO, K_2_O, and Na_2_O.

### Influence of the temperature on the property of ceramsite

As verified above, the ceramsite prepared at a high temperature of 1200°C displays a melting surface and expanded bubbles due to the slight addition of the fluxing agent Na_2_O. Therefore, a lower temperature to prepare ceramsite was investigated in order to reduce the sintering temperature and verify the influence of temperature on the property of the prepared ceramsite.

**FIGURE 7:**
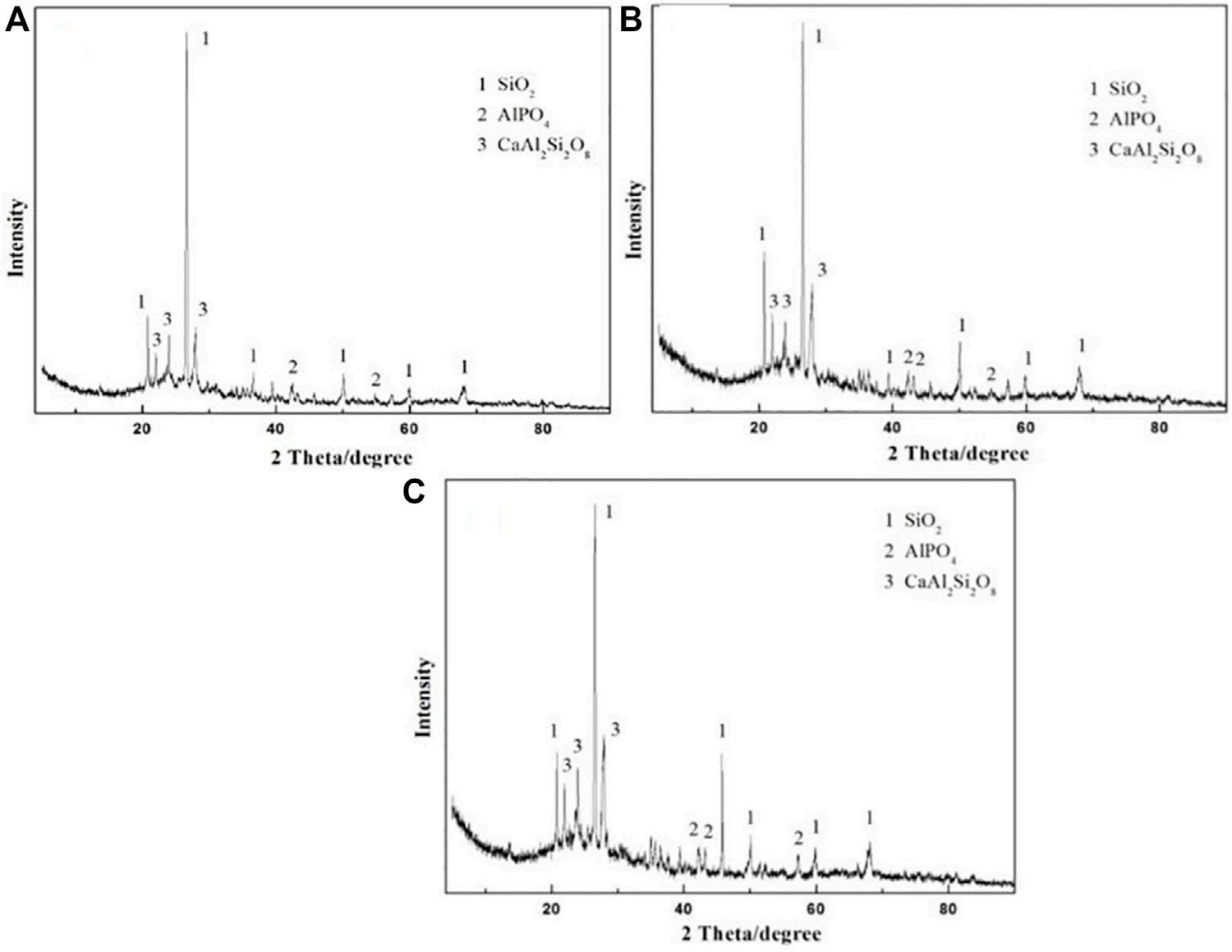
XRD pattern of the prepared ceramsite at different temperatures: **(A)** 1150°C, **(B)** 1175°C, and **(C)** 1200°C.

The XRD pattern of the prepared ceramsites at different temperatures from 1150^o^C to 1200°C is displayed in Figure 7. The main crystalline peaks identified in the prepared ceramsite are attributed to SiO_2_, AlPO_4_, and CaAl_2_Si_2_O_8_, which is almost the same even though a different temperature is applied. The peak of CaAl_2_Si_2_O_8_ becomes relatively stronger as the temperature increases.

**FIGURE 8:**
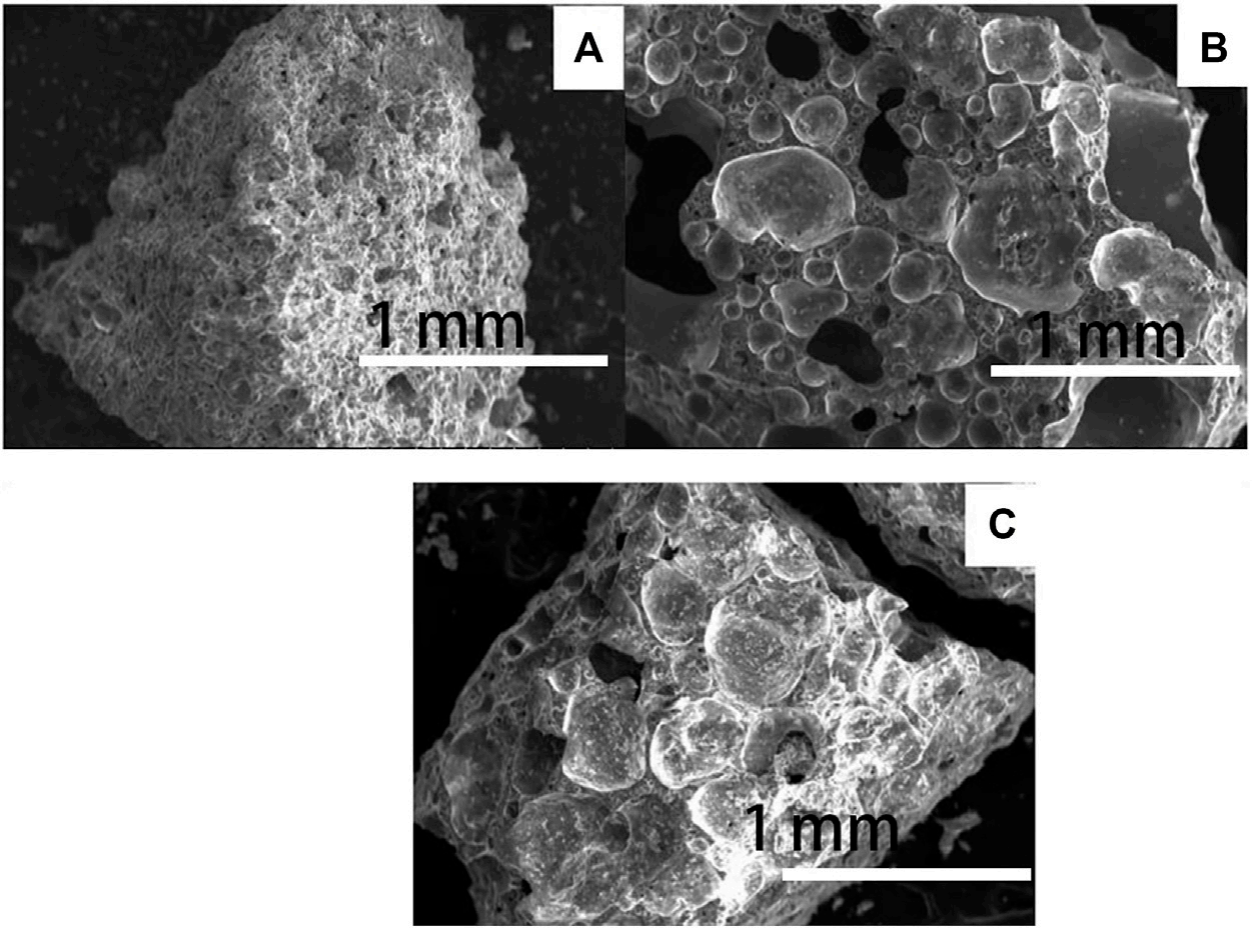
SEM image of the prepared ceramsite at different temperatures: **(A)** 1150°C, **(B)** 1175°C, and **(C)** 1200°C.

Figure 8 shows the microstructure of the cross-section of the prepared ceramsite at different temperatures. When the sintering temperature is 1150°C, some pores are formed; their perimeter is not smooth, and the bubble is not shaped. As the sintering temperature increases to 1175°C, the bubbles form evidently and their perimeter is smooth, as shown in Figure 8B. As the preparation temperature further increases to 1200°C, the bubble size becomes larger in general. Therefore, the high temperatures of 1175^o^C and 1200°C is necessary to prepare the sintering-expanded ceramsite.

### Influence of the pore-forming agent on the property of ceramsite

As above verified, the ceramsite prepared with the addition of the appropriate amount of the fluxing agent displays a melting surface and bubble formation. However, the generated bubble is not sufficiently big enough. According to the literature (Liao and Huang, 2012), Fe_2_O_3_ can increase the size of the ceramsite bubbles due to the formation of CO or CO_2_ gas from the chemical reactions between Fe_2_O_3_ and carbon.

**FIGURE 9:**
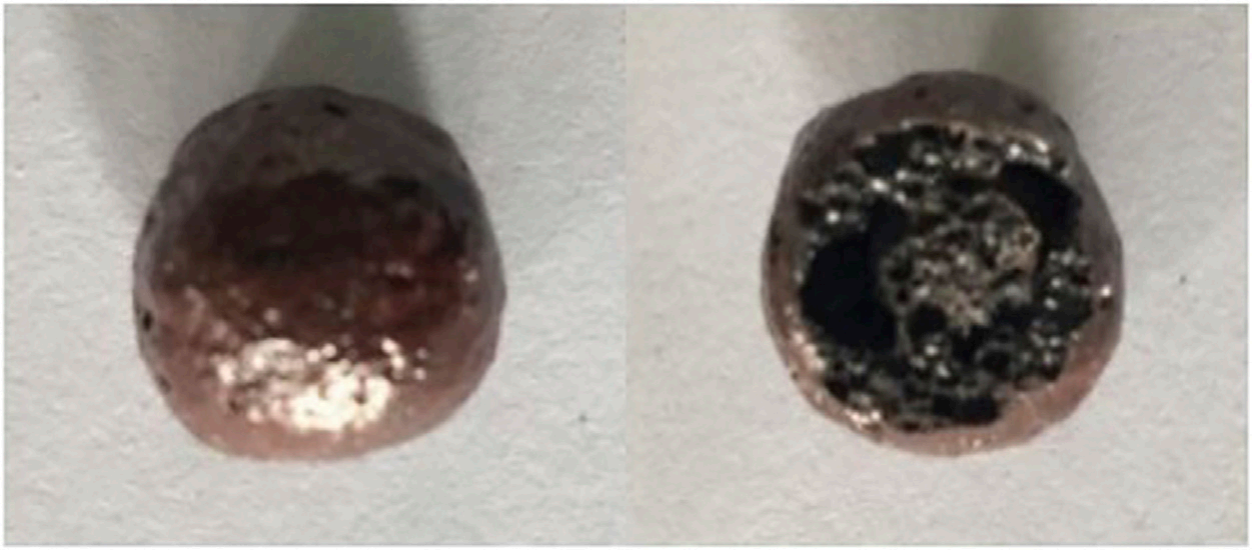
Photo of the prepared ceramsite with the addition of pore-forming agent Fe_2_O_3_ at 1200°C for 15 min.

**FIGURE 10:**
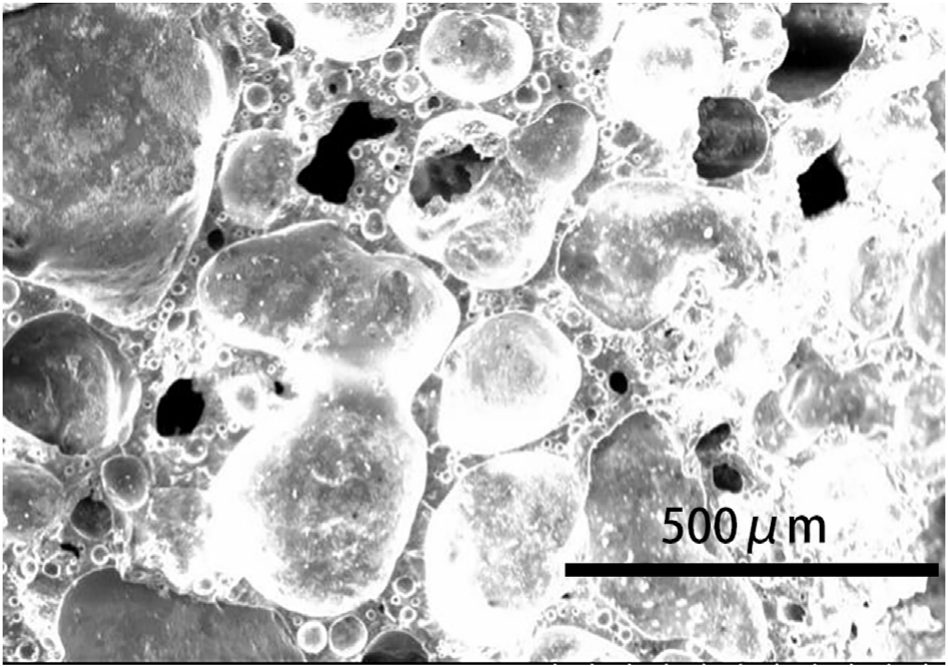
SEM image of the prepared ceramsite with the addition of pore-forming agent Fe_2_O_3_ at 1200°C for 15 min.

**FIGURE 11:**
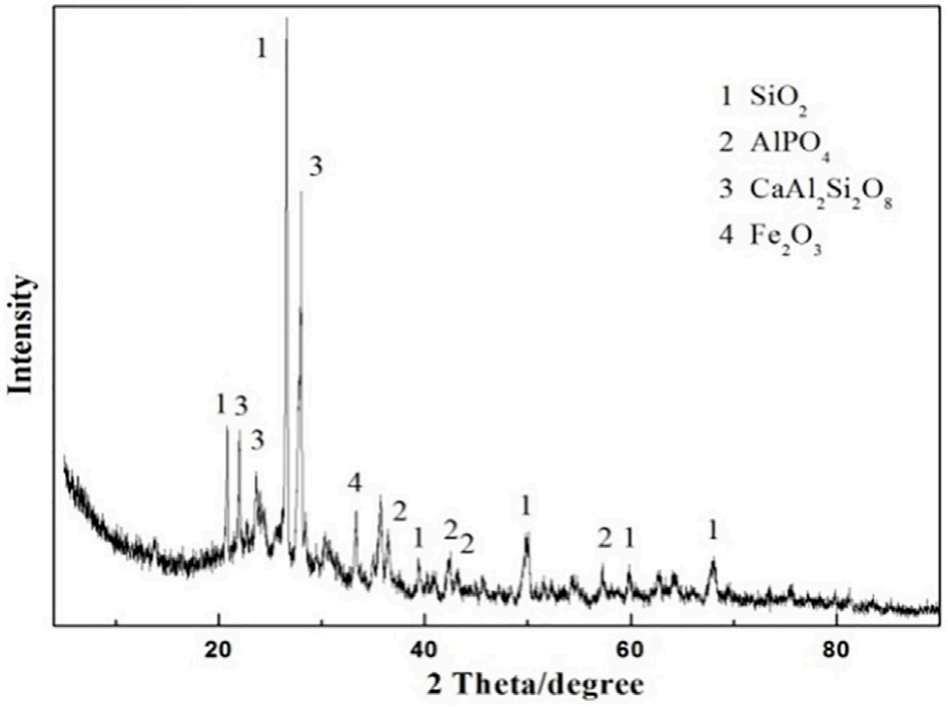
XRD pattern of the prepared ceramsite with the addition of pore-forming agent Fe_2_O_3_ at 1200°C for 15 min.

Therefore, the influence of the pore-forming agent on the property of ceramsite was investigated by the addition of the pore-forming agent Fe_2_O_3_ in order to increase the size of the bubbles generated during the sintering. The amount of Fe_2_O_3_ addition is set to be 4 wt% of the total weight of the original composition. The photo of the prepared ceramsite is shown in Figure 9, which displays a dark red color different from that of the ceramsite without the addition of a pore-forming agent. Also, a slight addition of Fe_2_O_3_ could obviously increase the bubble size of the ceramsite, whose size can be as big as about 4 mm. The SEM image of the generated bubble is displayed in Figure 10, which is relatively larger than that of the ceramsite prepared without the addition of the pore-forming agent. The density of the prepared ceramsite is about 0.95 g/cm^3^, smaller than that prepared without the addition of a pore-forming agent due to the increased size of the bubbles. The XRD pattern of the as-prepared ceramsite is shown in Figure 11, which shows that the main diffraction peaks are attributed to SiO_2_, AlPO_4_, CaAl_2_Si_2_O_8_, and Fe_2_O_3_.

### Leaching characteristics of heavy metals

As known, excess sludge contains some amount of organic matter and heavy metals, which is why it is considered hazardous solid waste in most cases. When the excess sludge is used to prepare ceramsite, the organic matter is completely destroyed due to the high temperature employed to prepare ceramsites during sintering. The hazardous factor of the ceramsite product is the heavy metal leaching toxicity. Therefore, the leaching toxicity of the original dried excess sludge and the as-prepared ceramsite is investigated according to China identification standards for hazardous wastes—identification for extraction toxicity (GB5085, 2007; HJ/T299, 2007). The investigated samples are as follows: the dried excess sludge, the ceramsite prepared from the mixture of excess sludge and clay, the ceramsite prepared with the addition of fluxing agent, and the ceramsite prepared with the addition of pore-forming agent. As seen in Table 2, the leaching toxicity of the heavy metals, including Ni, Cd, Pb, Cr, Cu, and V, in the dried excess sludge is high, although the leaching toxicity of the heavy metals is still lower than that of China Standard. When the excess sludge is used to prepare ceramsite, the leaching toxicity of the heavy metals in the prepared ceramsite decreases to a large extent. For instance, the leaching toxicity of the heavy metal Pb decreases to 0.01 mg/L, far lower than that of the dried excess sludge, which means the heavy metals are fixed in the ceramsite. The heavy metal leaching concentration decreases due to the combined effect of physical coating and chemical binding. This is also supported by the SEM image in Figure 6, which shows a dense structure due to the melting phenomenon which can prevent the leaching of heavy metals.

**TABLE 2:** Leaching toxicity of heavy metals of the dried excess sludge and the typically prepared ceramsite (mg/L).

element	Ni	Cd	Pb	Cr	Cu	V
Dried excess sludge	0.31	0.072	0.37	1.09	35.60	4.98
Ceramsite prepared from the mixture of excess sludge and clay	0.03	0.01	0	0.01	6.2	0.4
Ceramsite prepared with the addition of fluxing agent	0	0	0.01	0	1.9	0.21
Ceramsite prepared with the addition of a pore-forming agent	0.08	0	0	0.16	3.6	0.16
Identification Standard for hazardous wastes (GB5085.3-1996)	5	1	5	15	100	-

## Conclusion

The petrochemical excess sludge is applied to prepare ceramsite. The properties of ceramsite are primarily influenced by the addition of fluxing agent and the sintering temperature. The dried excess sludge cannot be alone adopted to prepare ceramsite due to the high content of the volatile component. The sintering-expanded ceramsite is not prepared when the dried excess sludge is mixed with clay. The slight addition of fluxing agent, Na_2_O, can reduce the melting temperature and prepare the sintering-expanded ceramsite, while an excess amount of fluxing agent can be detrimental to the preparation of ceramsite. Furthermore, the slight addition of a pore-forming agent, Fe_2_O_3_, can improve the bubble size and therefore reduce the bulk density of ceramsite. A temperature of 1200°C is necessary in order to prepare ceramsite, while a low temperature cannot expand ceramsite. In short, the optimal conditions for the preparation of ceramsite are as follows: dried excess sludge, 20 wt%; clay 71, wt%; Na_2_O, 5 wt%; and Fe_2_O_3_, 4 wt%. The leaching toxicity of the heavy metals is greatly reduced when the excess sludge is subject to sintering. Also, the leaching concentrations of the sintered products are all in compliance with China Identification Standard for hazardous wastes. Based on the abovementioned study, the petrochemical excess sludge can be used to prepare ceramsite in a resourceful way.

## Data Availability

The original contributions presented in the study are included in the article/supplementary material; further inquiries can be directed to the corresponding author.
